# Thirty‐two cats with effusive or non‐effusive feline infectious peritonitis treated with a combination of remdesivir and GS‐441524

**DOI:** 10.1111/jvim.16804

**Published:** 2023-07-04

**Authors:** Jodie Green, Harriet Syme, Sarah Tayler

**Affiliations:** ^1^ Royal Veterinary College Hatfield UK

**Keywords:** coronavirus, feline, FIP, microbiology, neurology, viral

## Abstract

**Background:**

GS‐441524 has been successfully used to treat feline infectious peritonitis (FIP) in cats. However, the use of its prodrug, remdesivir, in combination with a PO GS‐441524 containing product for the treatment of FIP has not yet been described.

**Objectives:**

Describe treatment protocols, response to treatment and outcomes in cats with FIP treated with a combination of PO GS‐441524 and injectable remdesivir.

**Animals:**

Thirty‐two client‐owned cats diagnosed with effusive or non‐effusive FIP including those with ocular and neurological involvement.

**Methods:**

Cats diagnosed with FIP at a single university hospital between August 2021 and July 2022 were included. Variables were recorded from time of diagnosis, and subsequent follow‐up information was obtained from the records of referring veterinarians. All surviving cats were observed for the entire 12‐week treatment period.

**Results:**

Cats received treatment with different combinations of IV remdesivir, SC remdesivir, and PO GS‐441524 at a median (range) dosage of 15 (10‐20) mg/kg. Clinical response to treatment was observed in 28 of 32 cats (87.5%) in a median (range) of 2 (1‐5) days. Twenty‐six of 32 cats (81.3%) were alive and in clinical and biochemical remission at the end of the 12‐week treatment period. Six of 32 cats (18.8%) died or were euthanized during treatment with 4 of the 6 cats (66%) dying within 3 days of starting treatment.

**Conclusions:**

We describe the effective use of injectable remdesivir and PO GS‐441524 for the treatment of FIP in cats. Success occurred using different treatment protocols and with different presentations of FIP including cats with ocular and neurological involvement.

**Abbreviations**
ALT: alanine transferase
BSH: British short hair
DSH: domestic short hair
FIP: feline infectious peritonitis
NIBP: non‐invasive blood pressure
RI: reference interval
SARS: severe acute respiratory syndrome

## 1 INTRODUCTION

Feline infectious peritonitis (FIP) is caused by a mutated biotype of the ubiquitous feline enteric coronavirus.^1^ Until recently it carried a grave prognosis because of a lack of efficacious treatments.^2^ In humans, emerging infectious diseases caused by RNA viruses such as Ebola and severe acute respiratory syndrome (SARS) have accelerated research into broad spectrum anti‐viral drugs including GS‐441524 and its prodrug remdesivir (GS‐5734).^3^, ^4^, ^5^

GS‐441524 is a nucleoside analog which prevents viral RNA replication by causing premature termination of viral RNA synthesis.^4^ Remdesivir is monophosphate prodrug of GS‐441524 which improves cellular penetration of the parent nucleoside.^5^ GS‐441524 and remdesivir are effective at inhibiting different species of coronavirus in tissue culture and in experimental animal models.^3^, ^4^, ^5^ GS‐441524 was found to successfully inhibit FIP virus replication in feline macrophages, and preliminary studies using GS‐441524 for the treatment of FIP in cats have shown promising results.^2^, ^6^, ^7^, ^8^, ^9^

Injectable GS‐441524 has been successfully used for the treatment of effusive and non‐effusive FIP in 25 of 31 cats treated with daily SC injections at a dosage of 2 to 4 mg/kg.^7^ Adverse effects were minimal and mostly attributable to injection site reactions. Disease relapse occurred in 8 of 26 cats within the first 3 to 84 days of treatment and was associated with lower treatment doses or treatment interruptions.^7^ Higher dosages of 5 to 10 mg/kg also have been effective in treating 3 of 4 cats with neurological FIP.^8^ A PO multi‐component drug (Xraphconn) containing GS‐441524 was used to successfully treat 18 cats with FIP with minimal adverse effects.^9^ However, neither of these drug formulations are licensed, nor legally accessible, for veterinary use in the United Kingdom (UK).

After the onset of the COVID‐19 pandemic, remdesivir was granted provisional licensing for the treatment of SARS‐Cov2 in humans in 2020.^5^, ^10^, ^11^, ^12^ As a result, access to injectable remdesivir for FIP treatment through a UK based compounding pharmaceuticals company became possible in August 2021 via the veterinary licensing cascade. This was followed by access to a PO formulation of GS‐441524 3 months later. However, no studies have yet evaluated the combined use of injectable remdesivir and PO GS‐441524 for the treatment of FIP in cats. Similarly, dose recommendations for remdesivir and PO GS‐441524 so far have been based on in vitro studies, experimental and limited field studies using injectable GS‐441524 and anecdotal evidence.^2^, ^6^, ^7^, ^8^, ^9^, ^13^, ^14^ Little therefore is known about optimal treatment doses for different disease presentations, treatment length and adverse effects with injectable remdesivir and PO GS‐441524.

Ours is a retrospective, observational case series describing treatment protocols, adverse effects, treatment response including clinical and biochemical changes, outcomes and survival rates in 32 cats with FIP treated with a combination of PO GS‐441524, injectable remdesivir or both. In our study, cats are described as achieving remission from FIP if they had resolution of all clinical signs and biochemical abnormalities by the end of the observed 12‐week treatment period. We aimed to describe treatment protocols and outcomes in cases of both effusive and non‐effusive FIP as well as cats that presented with ocular and neurological involvement.

## 2 MATERIALS AND METHODS

Ours was a retrospective study performed at the Queen Mother Hospital for Animals, Royal Veterinary College (London, UK) between August 2021 and July 2022. Ethical approval was granted by the institution's Ethical Review Board (URN SR2021‐0208). Cats were included if a diagnosis of FIP was made by a board‐certified small animal internal medicine specialist or a resident working under their supervision and the cat received treatment with either remdesivir or GS‐441524. A minimum follow‐up period of 12 weeks was required for all cats included, unless they were euthanized or died before the end of the follow‐up period. A treatment period of 12 weeks was used for all surviving cats based on previous studies.^2^, ^7^, ^13^ All follow‐up data was acquired from subsequent visits or from records of referring veterinarians.

### 2.1 Data collection

#### 2.1.1 Signalment and presenting signs

The following information was collected and recorded for all cats: weight, age, breed, sex and neuter status, body condition score, presenting signs and days from first clinical signs to diagnosis. Clinical examination findings were recorded including ocular examination by a board‐certified ophthalmologist or resident under their direct supervision and neurological findings by a board‐certified neurologist or resident under their direct supervision. All cats were classified as having effusive or non‐effusive FIP based on the presence or absence of pleural or peritoneal effusion or both on point‐of‐care ultrasound examination or other imaging modalities.

#### 2.1.2 Diagnosis

Diagnosis was based on a combination of signalment, clinical signs, hematologic findings, serum biochemistry, imaging, and cytologic examination. Coronavirus RT‐PCR on effusions or lymph node aspirates, immunocytochemistry and immunohistochemistry were considered desirable but not essential for diagnosis. All cases were reviewed by 2 authors—a resident in small animal internal medicine (JG) and a boarded‐certified small animal internal medicine specialist (ST). Cats were excluded if investigations were considered inadequate to support a diagnosis of FIP.^15^

#### 2.1.3 Treatment

The following information was recorded for all cats: dose of IV remdesivir treatment, number of days of IV remdesivir treatment, number of days of SC remdesivir treatment, dose of SC remdesivir treatment, number of days from diagnosis to starting treatment, dose of PO GS‐441524, number of days of PO GS‐441524 treatment, duration of total treatment course, changes to doses and treatment adverse effects. For cats with effusive FIP, the following was recorded: number of thoracocentesis procedures required before and after starting treatment and number of days until effusion improved or resolved. For all cats, days from first treatment to first clinical improvement, days from presentation to hospital discharge, concurrent medications, comorbidities, and procedures including sedation and general anesthesia were recorded.

#### 2.1.4 Follow‐up

Repeat clinical examination, hematology, and serum biochemistry were performed at 4, 8, and 12 weeks after starting treatment. For all cats, complications of treatment and signs of clinical relapse were recorded. For the cats that died or were euthanized, necropsy examination was available for 3 of 6 cats.

### 2.2 Statistics

Data collection, checking, and cleaning were performed using Microsoft Excel (2021). Categorical data were summarized with count and percentage. Data for continuous variables are reported as median (range). The data was imported into IBM SPSS (Statistical Product and Service Solutions) version 28 statistical software for analysis. Statistical analyses were carried out to identify whether there was a significant increase in albumin: globulin ratios for cats with albumin: globulin ratios performed at the following time points: from 0 to 4 weeks, from 4 to 8 weeks, and from 8 to 12 weeks after starting treatment. Shapiro‐Wilks test for normality was carried out on the difference between paired values. Parametric data was compared using a paired *t* test, and a paired Wilcoxon signed‐rank test was used for non‐parametric data. Significance was defined as *P* < .05 for all analyses.

## 3 RESULTS

### 3.1 Signalment

Thirty‐two cats were included: 12 British Short Hair (BSH), 5 Siberian, 5 Ragdoll, 3 Bengal, 3 Domestic Short Hair (DSH), 2 Maine Coon, 1 Turkish Vann and 1 Domestic Long Hair. There were 6 intact males, 16 neutered males, 4 intact females and 6 spayed females. Age at presentation was 7.5 (4‐72) months, body weight was 2.9 (1.14‐6) kg, and body condition score was 3 (1‐7) out of 9.

### 3.2 Clinical presentation

**FIGURE 1:**
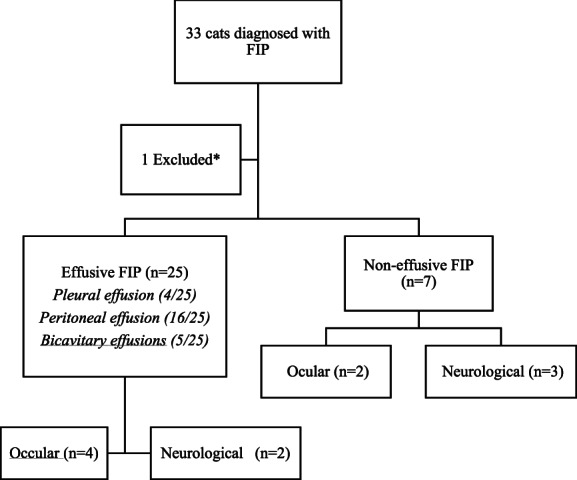
Diagram reflecting the different presentations of feline infectious peritonitis for the 32 cats included in this study. *One cat was excluded due to the owner obtaining non‐licensed GS‐441524 4 weeks into the treatment period.

Characteristics of 32 cats diagnosed with FIP are summarized in Figure 1 and Table S1.

#### 3.2.1 Presenting signs

The most common presenting signs for all 32 cats were lethargy (32/32; 100%), hyporexia or anorexia (28/32; 87.5%), weight loss or poor weight gain (19/32; 59.4%), abdominal distension (21/32; 65.6%), diarrhea (7/32; 21.9%), dyspnea (5/32; 15.6%), ataxia (5/32; 15.6%), and icterus (4/32; 12.5%); time from first clinical signs to presentation was 9 (3‐122) days.

#### 3.2.2 Effusive and non‐effusive disease

Twenty‐five cats presented with effusive FIP. Four cats had pleural effusion, 16 cats had peritoneal effusion and 5 cats had bi‐cavitary effusions. Seven cats presented with non‐effusive FIP.

#### 3.2.3 Ocular signs

Six cats had evidence of ocular involvement. Four cats had chorioretinitis, 1 cat had left anterior uveitis with green iridial discoloration, 1 cat had bilateral anterior uveitis with keratoprecipitates, and 1 cat had bilateral uveitis and chorioretinitis.

#### 3.2.4 Neurological signs

Five cats presented with neurological signs. All 5 cats had marked obtundation, 2 cats had proprioceptive ataxia, 1 cat had cerebellar ataxia, and 2 cats had vestibular ataxia.

### 3.3 Diagnostic testing

Hematologic and biochemical findings are summarized in Table 1 and Table S1. Diagnostic imaging, cytologic, histopathologic, and immunocytochemistry findings are summarized in Table 2. Five of 16 cats (31%) that had non‐invasive blood pressure (NIBP) measured at presentation were hypotensive (Doppler NIBP <80 mmHg).

**TABLE 1:** Table showing biochemical and hematological findings in 32 cats with feline infectious peritonitis at the time of diagnosis.

	Number of cats (out of 32)	%
Biochemistry		
Hyperbilirubinemia (>5.1 mmoL/L)	27	84
Hypoalbuminemia (<26.3 g/L)	31	97
Hyperglobulinemia (>45 g/L)	23	72
Albumin: Globulin Ratio < 0.4	23	72
Albumin: Globulin Ratio 0.4‐0.6	7	22
Albumin: Globulin Ratio > 0.6	2	6
Hypoglycemia (<3.1 mmol/L)	5	15
Hematology		
Anemia (PCV < 24%)	10	31
Neutrophilia (>12.5 × 10^9^/L)	10	31
Lymphopenia (<1.5 × 10^9^/L)	26	81

**TABLE 2:** Table showing diagnostic findings in 32 cats with feline infectious peritonitis.

Diagnostics	Number of cats (out of 32)	Findings	Number of cats (out of 32)
Imaging			
Thoracic radiographs	5	Pleural effusion Abdominal lymphadenopathy Nephropathy Colonic wall thickening Peritoneal effusion Generalized ventricular dilation and ependymal contrast enhancement	9 16 5 5 21 1
CT	2		
Abdominal ultrasound	22		
MRI	1		
Cytology			
Fluid (peritoneal/pleural)	24	Protein rich transudate with pyogranulomatous inflammation Neutrophilic exudate	23 1
Lymph node	10	Pyogranulomatous inflammation	10
Further diagnostics			
Coronavirus antibody titres (fluid or blood)	13	Positive	13
Coronavirus RT‐PCR (fluid or lymph node)	6	Positive	6
Immunocytochemistry/immunohistochemistry (fluid or lymph node)	5	Positive Negative	4 1

### 3.4 Treatment

**FIGURE 2:**
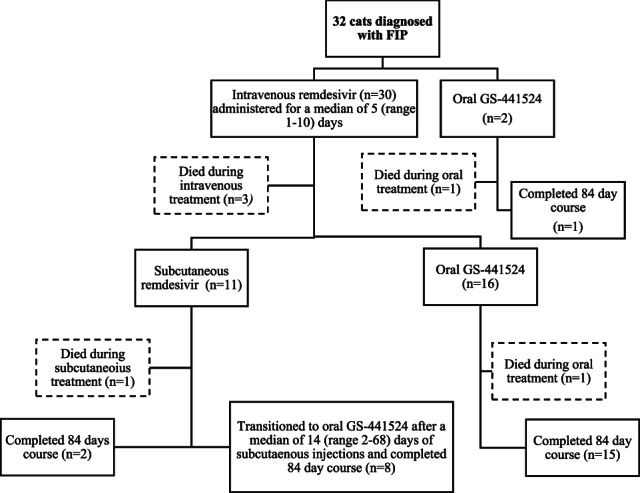
Diagram depicting the different treatment courses with remdesivir and GS‐441524 for 32 cats diagnosed with effusive and non‐effusive feline infectious peritonitis.

Treatment protocols are summarized in Figure 2 and Table S1.

#### 3.4.1 Intravenous remdesivir treatment

Thirty of 32 cats commenced treatment with IV remdesivir. Median (range) time from admission to starting treatment was 1 (0‐9) days and duration of IV remdesivir treatment was 5 (1‐10) days. For all cats, remdesivir was diluted 50 : 50 with saline and given as a constant rate infusion over 1 h. Overall starting dosage was 15 (10‐20) mg/kg. Nine cats (mainly those with stable effusive FIP) were given a starting dosage of 10 mg/kg once daily, 15 cats 15 mg/kg and 6 cats 20 mg/kg. Higher doses were used for cats with neurological FIP, those with ocular involvement or those with unstable effusive FIP, including those with hypotension, hypoglycemia, and severe pleural effusion.

#### 3.4.2 Subcutaneous remdesivir treatment

Eleven cats received SC remdesivir treatment after initial IV remdesivir treatment. Two cats (1 effusive FIP and 1 non‐effusive, neurological FIP) received only SC remdesivir treatment after IV treatment and completed the 84‐day treatment course. One cat received 2 doses of SC remdesivir before euthanasia. Eight cats received SC injections of remdesivir for a median (range) of 14 (2‐68) days before transitioning onto an equivalent dose of PO GS‐441524 to complete the 84‐day treatment course. Median (range) dosage of SC remdesivir was 10 (10‐15) mg/kg.

#### 3.4.3 Oral GS‐441524 treatment

Two cats never received IV remdesivir and were started on PO GS‐441524 from initial presentation. One cat was markedly azotemic on presentation and was euthanized 48 h after beginning treatment because of worsening acute kidney injury. The second cat completed an 84‐day course of PO GS‐441524. Sixteen cats transitioned immediately from IV remdesivir to an equivalent dose of PO GS‐441524 after 5 (5‐10) days and completed the entire 84‐day course. The dosage of PO GS‐441524 was 15 (10‐20) mg/kg.

### 3.5 Concurrent medications and co‐morbidities

#### 3.5.1 Concurrent medications

Concurrent medications included maropitant (n = 15), ondansetron (n = 12), mirtazapine (n = 10), dextrose supplementation (n = 5), buprenorphine (n = 3), antibiotics (n = 3), furosemide (n = 1), norepinephrine (n = 2) hydrocortisone (n = 2), methadone (n = 1), packed red blood cell transfusion (n = 2), clopidogrel (n = 1), and prednisolone (n = 1). All cats with ocular involvement received topical medications including cyclopentolate hydrochloride (n = 2), ketorolac (0.5%, Acular; n = 2) topical dexamethasone (0.1%, Maxidex; n = 2), and latanoprost (n = 1).

#### 3.5.2 Co‐morbidities

One cat developed congestive heart failure while hospitalized and echocardiography was suggestive of hypertrophic cardiomyopathy phenotype. One cat developed phlebitis and necrosis of the right cephalic skin at a previous IV catheter site and required multiple general anesthetic administrations for surgical debridement and primary closure. One cat underwent sedation for nasogastric feeding tube placement and developed suspected aspiration pneumonia. One cat that received IV meloxicam off‐license for 3 days before presentation developed worsening acute kidney injury within 48 h of presentation.

### 3.6 Adverse effects

No clinically relevant adverse effects were observed with IV remdesivir or from PO GS‐441524 treatment. Seven cats reacted adversely to SC injections with immediate pain reactions. All 7 of these cats received gabapentin before SC injection and 10 cats received topical local anesthetic (EMLA 5%, AstraZeneca) at the injection site to facilitate administration. Two cats developed local skin reactions that resolved without intervention.

### 3.7 Outcome

#### 3.7.1 Clinical response

Clinical response was documented in 28 of 32 cats (87.5%) in 2 (1‐5) days. Clinical response was recorded as improvement in demeanor (28/28; 100%), improvement in appetite (24/24;100%), resolution of pyrexia (20/20; 100%), and improvement in neurological signs (4/4; 100%). Eight of 9 (89%) cats with pleural effusion underwent thoracocentesis before starting treatment. Four of 9 cats (44%) required repeat thoracocentesis within 24 h of starting treatment and no cats required repeat thoracocentesis >24 h after starting treatment. All 9 cats had improvement in pleural effusion documented within 3 days of commencing treatment. Only 3 cats required >3 days of treatment before clinical response was documented. All 3 of these cats were Ragdolls and all suffered subsequent relapses in their conditions while still receiving treatment. Two of these cats were euthanized as a result.

#### 3.7.2 Survivors

Twenty‐eight of 32 cats (87.5%) survived to discharge from hospital. Time from admission to discharge was 7 (4‐19) days. Twenty‐six of 32 cats (81.3%) were still alive and considered to be in clinical remission at the end of the 12‐week treatment course. These included 4 of 5 cats (80%) that presented with neurological disease and 4 of 6 cats (67%) with ocular involvement. Follow‐up time after finishing treatment was 258 (151‐402) days and all cats were still alive at last follow‐up. One surviving cat (a Ragdoll with effusive and ocular FIP) with clinical signs that had initially improved substantially 4 days into treatment suffered a relapse of clinical signs consisting of pyrexia, anorexia, green irideal discoloration and anterior uveitis 7 days into treatment and 2 days after transitioning from IV to SC remdesivir at 15 mg/kg. This cat was returned to IV remdesivir at 20 mg/kg for 5 days before completing the 12‐week treatment period with 20 mg/kg PO GS‐441524. One cat (a DSH with effusive FIP) that survived to 12 weeks developed pyrexia and anorexia 2 days after finishing the initial treatment course. Clinical investigations were suspicious of a relapse, and the cat was started on a second 84‐day course of GS‐441524 at a dosage of 20 mg/kg (previously 10 mg/kg). The cat was in clinical remission after finishing the second 84‐day treatment course.

#### 3.7.3 Non survivors

Six of 32 cats (18.8%) died or were euthanized during treatment (2 BSH, 3 Ragdolls, and 1 Siberian). Four of 6 of these cats (67%) were hypoglycemic (blood glucose concentration [BG] < 3.5 mmol/L) on presentation and 3 of 4 of these cats (75%) were also hypotensive (Doppler NIBP <80 mmHg) on presentation. Four of 6 cats (66%) died or were euthanized within 3 days of starting treatment. One cat (BSH with effusive and ocular FIP) was euthanized 2 days into treatment because of condition severity. Necropsy examination confirmed the diagnosis of FIP. One cat (Siberian with effusive FIP) was euthanized after 2 days because of worsening acute kidney injury; this cat had been receiving IV meloxicam for 3 days before referral and was azotemic before starting treatment. One cat (BSH with effusive and neurological disease) with marked hypoglycemia and hypotension suffered cardiopulmonary arrest under anesthesia for placement of a jugular catheter 1 day after starting treatment. Necropsy examination confirmed the diagnosis of FIP. One cat (a Ragdoll with effusive and ocular FIP) deteriorated substantially within 24 h of admission with refractory hypotension and hypoglycemia and subsequently was euthanized.

Two cats were euthanized after discharge at 12 and 13 days into treatment. One cat (Ragdoll with non‐effusive FIP) showed an initial response to 15 mg/kg IV and SC remdesivir treatment and was discharged after 7 days. This cat developed thoracolumbar myelopathy within 24 h of returning home. The cat was restarted on IV remdesivir at 20 mg/kg for 5 days with no clinical improvement and was euthanized 12 days into treatment. Necropsy examination confirmed the clinical suspicion of progressive FIP within the central nervous system. One cat (Ragdoll with effusive FIP) showed a slow clinical response to treatment with first signs of improvement after 5 days. This cat subsequently developed suspected aspiration pneumonia after placement of a nasogastric tube and was discharged 12 days into treatment. The cat suffered from a seizure 24 h after discharge with suspected progressive neurological FIP and was subsequently euthanized.

Excluding the 4 cats that were euthanized within 3 days of starting treatment, 26 of 28 cats (92%) were still alive at the end of the 12‐week treatment period.

### 3.8 Follow‐up

Follow‐up hematology and serum biochemistry results were available for 25 of the 26 surviving cats. Sixteen cats had follow‐up laboratory work performed at 4 weeks, 15 cats at 8 weeks and 18 cats at 12 weeks. No hematologic abnormalities were apparent at 4, 8, or 12 weeks. Serum biochemical abnormalities documented at each time point are summarized in Table 3 and in Table S1.

**TABLE 3:** Table showing biochemical abnormalities present in cats with feline infectious peritonitis at time points 0, 4, 8, and 12 weeks into treatment with a combination of injectable remdesivir and oral GS‐441524.

	Number of cats		
Biochemical abnormality	Week 4	Week 8	Week 12
Hyperglobulinemia (>45 g/L)	8/16 (50%)	3/15 (20%)	0/18 (0%)
Hyperbilirubinemia (>5.1 mmol/L)	5/16 (31%)	2/15 (13%)	0/18 (0%)
Hypoalbuminemia (<26.3 g/L)	2/16 (12.5%)	0/15 (0%)	0/18 (0%)
ALT >60 U/L	10/16 (62%)	8/15 (53%)	6/18 (33%)

Of the 25 cats, 8 cats (32.0%) had an increase in alanine aminotransferase (ALT) activity >2‐fold from baseline and above the reference interval (>60 U/L) at 4, 8, or 12 weeks into treatment. Two cats (8.0%) experienced transient increases in serum creatinine concentration of >30% and > 150 μmol/L (reference interval [RI], 50‐177 μmol/L).

Follow‐up echocardiography was performed 4 weeks into treatment and 4 weeks after finishing a 12‐week treatment course in 1 cat (DSH with effusive FIP) diagnosed with hypertrophic cardiomyopathy phenotype and congestive heart failure at presentation. Phenotype stage B2 was documented at 4 weeks but all phenotypical abnormalities had resolved by 12 weeks.

#### 3.8.1 Serum albumin: globulin ratios

**FIGURE 3:**
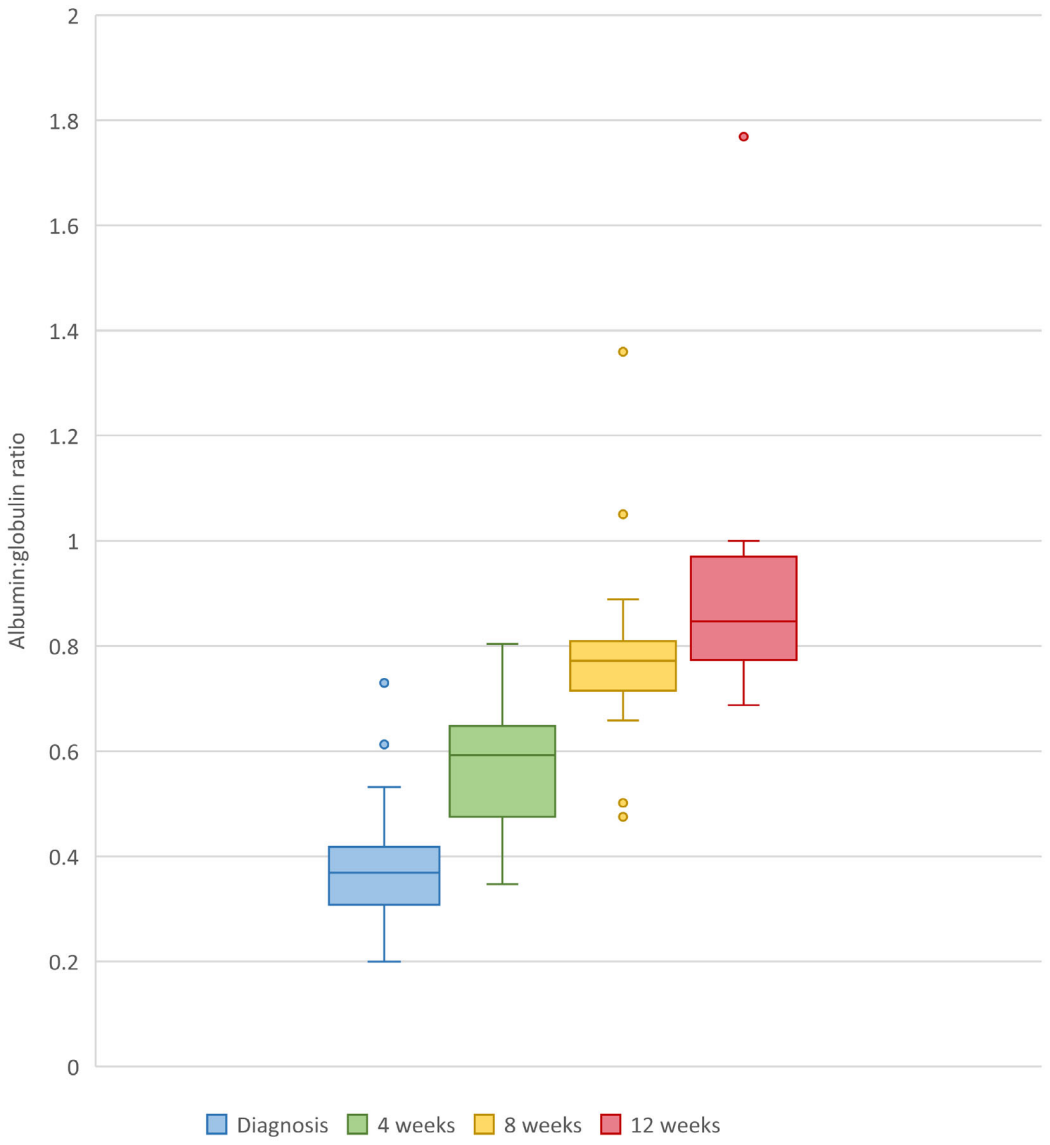
Box and whisker plot depicting albumin: globulin ratios of 25 cats with feline infectious peritonitis at 4, 8, and 12 weeks into treatment with remdesivir and/or GS441524.

Median serum albumin: globulin ratios continued to increase for all cats at 4, 8, and 12 weeks into treatment (Figure 3). A significant increase in albumin: globulin ratios occurred for cats with albumin: globulin ratios performed at both time points from week 0 to week 4 (*P* < .01), from week 4 to week 8 (*P* < .01) and from week 8 to week 12 (*P* = .01).

## 4 DISCUSSION

Our case series demonstrates the effective use of injectable remdesivir and PO GS‐441524 for the treatment of naturally‐occurring FIP. When cats survived the initial 3 days of treatment, outcome was considered excellent, with 26 of 28 cats (92.8%) still alive and in clinical remission at the end of the 12‐week treatment period. This outcome is consistent with previous success rates reported with SC GS‐441524 treatment.^2^, ^7^, ^8^ Our study also supports client‐reported survey results on a PO GS‐441524‐like compound for the successful treatment of FIP.^16^

Herein, we describe treatment protocols for FIP using a combination of IV remdesivir, SC remdesivir and PO GS‐441524. No studies have yet assessed optimal dosing of remdesivir, the prodrug of GS‐441524, for the treatment of FIP in cats. Doses of remdesivir in our study therefore were extrapolated from dosages used in human medicine, from previous studies on GS‐441524 and from anecdotal evidence.^2^, ^7^, ^8^, ^9^, ^14^, ^17^ Optimal dosing of PO GS‐441524 also has not yet been established although dosages of 5 to 10 mg/kg for 12 weeks have been used successfully.^9^, ^16^ Doses equivalent to the doses used for IV remdesivir were used in our study because of the poor oral bioavailability of GS‐441524 identified in other species despite its molecular weight being roughly half that of remdesivir.^18^ Additional pharmacokinetic studies are needed however to determine the optimal dosing of both remdesivir and PO GS‐441524 for cats with FIP.

Because of the small number of cats receiving each treatment protocol and the retrospective nature of our study, comparisons cannot be made among treatment protocols and conclusions cannot be drawn on associated treatment success. However, effective treatment did occur using all treatment combinations. Treatment protocols were decided on a case‐by‐case basis and were influenced by several factors. A PO formulation of GS‐441524 was not available for the period of August to November 2021 and, as a result, all cats were transitioned from IV to SC remdesivir. However, once available, cats were subsequently transitioned onto an equivalent dose of PO GS‐441524 after variable durations of SC treatment. Subcutaneous injections were associated with a larger financial burden, difficulties in administration, poor patient tolerance and adverse effects such as pain on injection. As a result, SC injections in many patients consisted only of a short course or were negated altogether. In contrast, PO GS‐44152 did not appear to be associated with clinically relevant complications and was well tolerated as has been reported previously.^9^ These findings also are consistent with previously reported owner perceptions that administration by injection was a major disadvantage in SC GS‐441524 treatment.^16^ Poor compliance with treatment previously has been associated with an increased risk of relapse and must be considered when designing treatment protocols.^7^ The success of combining IV remdesivir and PO GS‐441524 for the treatment of FIP in our study, in light of the disadvantages of SC remdesivir injection, therefore may lead to preferential use of PO GS‐441524 over SC remdesivir injections in the future.

Treatment success also occurred in our study with different presentations of FIP including cats with neurological and ocular involvement. Cases of neurological and ocular FIP require higher doses of injectable GS‐441524 because of poor drug access across the blood‐ocular and blood‐brain barriers.^2^, ^8^ Previous pharmacokinetic studies have shown that dosages of 10 mg/kg of GS‐441524 result in cerebrospinal fluid concentrations in healthy cats capable of causing partial viral inhibition in vitro.^2^ Cases of neurological FIP or ocular FIP in our study therefore were started on dosages of 15 to 20 mg/kg of remdesivir and PO GS‐441524 q24h. Four of 5 cats (80%) that presented with neurological disease and 4 of 6 cats (67%) with ocular involvement recovered fully. However, 2 cats (both Ragdolls) that had originally presented without neurological signs developed neurological signs after discharge >1 week into treatment and subsequently were euthanized. These findings suggest that neurological disease developed and progressed despite treatment in these cats. This phenomenon has been reported previously, but the cause of treatment failure is unknown.^7^ It is possible that sub‐therapeutic concentrations of GS‐441524 within the cerebrospinal fluid and the development of viral resistance may play contributory roles.^2^, ^7^, ^13^ Additional pharmacokinetic studies therefore are required to determine optimal remdesivir and GS‐441524 dosing in cases with ocular and neurological involvement.

In our study 6 of 32 cats did not survive the treatment period. Four of these cats (67%) were hypoglycemic on presentation, which may reflect disease severity and the presence of severe inflammatory response syndrome or sepsis. Unfortunately, necropsy examination and blood cultures were not available for all cats. All 4 cats died within 72 h of starting treatment. Mortality in our study however also was influenced by other contributing factors including financial constraints resulting in cats not being allowed sufficient time to respond to treatment. True rates of clinical response therefore may be higher than our case series suggests. Additional investigations are required to identify whether cats that do not respond are refractory to treatment because of the presence or development of viral resistance or because of severe and irreversible inflammatory changes.^7^, ^13^

In our study, 3 of 5 Ragdolls were euthanized because of their disease. Three of the 4 Ragdolls that survived more than 24 h demonstrated a delayed response to treatment and relapsed while receiving treatment, raising concerns for a poorer response in this breed. Only 1 Ragdoll in our study survived to 12 weeks without evidence for clinical relapse, however, there were too few Ragdolls in our study to determine if this breed is associated with poorer outcome, because this finding has not been reported in previous studies using GS‐441524. Future studies investigating breed‐specific differences in response to treatment in a larger cohort of cats are warranted to investigate this association further.

Follow‐up hematology and serum biochemistry results were available for 25 of 26 cats, but only 9 cats had blood tests performed at every time point, which likely was a consequence of financial constraints of the owners. In our study, albumin: globulin ratios continued to improve significantly at every time point. This observation supports previous findings that the albumin: globulin ratio may be a useful treatment monitoring marker.^7^ Furthermore, complete resolution of hyperbilirubinemia, hyperglobulinemia, and hypoalbuminemia only occurred 8 to 12 weeks into treatment in some cats. These findings, along with similar findings in previous studies, therefore may support a minimum treatment period of 12 weeks.^7^ Additional studies are needed to identify the most effective treatment duration for the different presentations of FIP using different treatment protocols.

In our study, 32% of the cats had more than a 2‐fold increase in ALT activity above the limit of the RI at 4, 8, or 12 weeks into treatment. This finding has been reported previously in cats treated with GS‐441524.^9^ In humans with COVID‐19, minor serum aminotransferase increases occur in patients treated with remdesivir without other evidence of hepatic injury, and resolve after cessation of treatment.^19^, ^20^ The cause of hepatopathy with remdesivir and GS‐441524 treatment is not understood, but may be the result of direct toxicity because of inhibition of mitochondrial RNA polymerase.^19^, ^20^

In our study follow‐up requirement was only the 12‐week treatment period. As a result, the frequency of relapse of clinical signs and the duration of sustained remission after finishing 12 weeks of treatment cannot be established. Additional studies are required to establish longterm follow‐up and outcome.

In conclusion, our case series demonstrates the effective use of injectable remdesivir and PO GS‐441524 for the treatment of effusive and non‐effusive FIP in cats. Success occurred using different treatment protocols, and with different presentations of FIP, including cats with ocular and neurological involvement. Additional prospective studies are required to identify the most effective treatment protocols for the different presentations of FIP, and to provide longterm follow‐up information after the end of treatment.

## CONFLICT OF INTEREST DECLARATION

United Kingdom based Specials Company BOVA contributed financially to the treatment of 1 cat in this study.

## OFF‐LABEL ANTIMICROBIAL DECLARATION

Authors declare no off‐label use of antimicrobials.

## INSTITUTIONAL ANIMAL CARE AND USE COMMITTEE (IACUC) OR OTHER APPROVAL DECLARATION

Approved by the Royal Veterinary College University, URN SR2021‐0208.

## HUMAN ETHICS APPROVAL DECLARATION

Authors declare human ethics approval was not needed for this study.

## Supporting information


**TABLE S1.** Table showing clinical findings of 32 cats diagnosed with Feline Infectious Peritonitis (FIP) treated with a combination of remdesivir and GS‐441524
